# Here Comes the Sun: Xanthophylls as Components of Plant Memory

**DOI:** 10.1111/ppl.71136

**Published:** 2026-10-01

**Authors:** José Ignacio García‐Plazaola, Miren Irati Arzac, Águeda M. González‐Rodríguez, Laura Díaz‐Jiménez, Alicia V. Perera‐Castro, Beatriz Fernández‐Marín

**Affiliations:** ^1^ Department Plant Biology and Ecology, Faculty of Sciences and Technology, Basque Center for Integrative and Human Health (EHU‐iBio) University of the Basque Country (EHU) Leioa Spain; ^2^ Plant Ecophysiology and Irrigation (ECOVER) Group Instituto de Recursos Naturales y Agrobiología de Sevilla (IRNAS‐CSIC) Sevilla Spain; ^3^ Department Botany, Ecology and Plant Physiology University of La Laguna (ULL) La Laguna Canary Islands Spain; ^4^ Department of Biology Universitat de les Illes Balears Palma Spain

## Abstract

It has been proposed that the de‐epoxidation state of the xanthophyll cycle (AZ/VAZ) represents a mechanism of light memory storage in plants as it depends on the amount of light previously received by the leaf, later affecting the photosynthetic responses by modulating the rate of non‐photochemical quenching (NPQ). In this study we examine what the time window is for which the cumulative photon dose (CumPFD) best explains AZ/VAZ. For this purpose, we developed a field experiment in a boreal forest in Finland on 17 species representing all major functional groups. In all species AZ/VAZ correlated more strongly with CumPFD than with instantaneous light intensity, supporting its role as a form of light memory. Besides AZ/VAZ was linked to photochemical efficiency, demonstrating that it influences current photosynthetic performance. However, the duration of this memory differed among species, with some (mosses, ferns, herbs and deciduous woody plants) where AZ/VAZ best reflected CumPFD of the previous 2–5 h (short‐term memory) and others (mainly evergreen species) where AZ/VAZ integrated CumPFD received for 10–15 h (long‐term memory). Overall, these results show that AZ/VAZ fulfills the concept of a memory mechanism. Additionally, they pinpoint that this form of storage is transient, varies with plant functional types, and generally vanishes within 24 h.

## 1 Introduction

Photosynthetic organisms have a “love/hate” relationship with light (Müller et al. 2001). On the one hand, they need light as the source of energy that fuels photosynthesis and, because of this, plants have evolved specific organs to maximize photon harvesting. On the other hand, when the light absorption surpasses the amount that can be metabolically used, the excess of energy can be channeled toward the production of reactive oxygen species, damaging organelles, cells and ultimately whole plants. To achieve a correct balance between energy use and protection, during the course of evolution plants have developed a sophisticated combination of protective mechanisms which operate at different levels of integration (for recent reviews see: Bassi and Dall'Osto 2021; van Amerongen and Croce 2025). Among them, the direct emission as heat of the energy absorbed by chlorophylls, the so‐called thermal energy dissipation, is the fastest, safest and most finely regulated response (Zuo 2025). The activation of this mechanism obviously implies a decrease in photochemical efficiency, as the energy of photons is dissipated as heat rather than being used for carbon fixation. Thus, this process can be easily identified by a decrease in the fluorescence yield of chlorophyll, the so‐called “non‐photochemical quenching”, abbreviated NPQ. In fact, the term NPQ is frequently employed as a synonym for thermal energy dissipation, although it strictly refers to its underlying effect on chlorophyll fluorescence.

NPQ is a complex mechanism that involves several components (Bassi and Dall'Osto 2021; Nilkens et al. 2010; van Amerongen and Croce 2025) which act in a coordinated way, providing a fine adjustment of the amount of energy that needs to be dissipated at each time, always taking into account the simultaneous estimation of the metabolic demands and the light energy inputs (Jahns and Holzwarth 2012; Zuo 2025). The activation of NPQ initially requires the formation of a trans‐thylakoid proton gradient generated by the onset of the light‐driven electron transport chain (Matuszynska et al. 2016). As this gradient is the force that allows ATP synthesis, its magnitude reflects the balance between energy production and metabolic demand, decreasing when ATP consumption is high. Therefore, the light‐induced proton (pH) gradient acts as the sensor of light energy excess, supporting the idea that PSII functions as a “photon counter” as originally proposed by Park et al. (1995, 1996). The generation of a ΔpH across thylakoid membranes causes an initial decrease of fluorescence yield, which represents the fastest component of NPQ, the so‐called qE, which is likely caused by conformational changes in the PSII antennae (Müller et al. 2001). Although the exact mechanism remains unclear, recent evidence suggests that qE is driven by a localized conformational switch in a few antenna complexes. This switch is triggered by PsbS (and/or LHSCR for bryophytes and algae), with zeaxanthin having an essential and potentially amplificative role (Pinnola 2019; van Amerongen and Croce 2025). The acidic conditions inside the thylakoid activate an enzyme, violaxanthin de‐epoxidase (VDE), which catalyzes the de‐epoxidation of the xanthophyll violaxanthin (V) to form zeaxanthin (Z), through the intermediate antheraxanthin (A; Goss et al. 2008). In darkness, when the ΔpH dissipates, the reverse reaction takes place, catalyzed by the zeaxanthin epoxidase (ZE), giving rise to the so‐called xanthophyll, or VAZ, cycle (Demmig‐Adams and Adams 1996). Thus, the presence of de‐epoxidised xanthophylls (A and Z) seems to amplify qE (Jahns and Holzwarth 2012) and contributes to NPQ even in the absence of ΔpH (qZ; Nilkens et al. 2010). The relative proportion of de‐epoxidised xanthophylls (A + Z)/(V + A + Z), also known as de‐epoxidation index or AZ/VAZ (Demmig‐Adams and Adams 1996), is the most frequently used expression to characterize the conversion state of the xanthophyll cycle (Esteban et al. 2015; Fernández‐Marín et al. 2021). Given its regulatory nature of thermal energy dissipation, the ratio AZ/VAZ usually correlates positively with NPQ and negatively with the maximum photochemical efficiency of PSII (*F*_v_/*F*_m_) and photosynthetic efficiency (Kromdijk et al. 2016). NPQ has other components: qI, involved in photoinhibitory quenching; qT, related to the state transitions; and qH, which includes sustained forms of photoprotective quenching (Ruban 2016; Wobbe et al. 2016; Zuo 2025; Malnoë 2018). In addition, some authors include qM, which is related to chloroplast movements, but does not represent a “genuine” mechanism of thermal dissipation (Cazzaniga et al. 2013).

Thus, as nicely reviewed by Jahns and Bethmann (2025), three main factors determine the amount of A + Z at a certain moment and at three different time scales: (i) the growth light conditions that determine the total amount of xanthophyll cycle pigments in the thylakoid membrane (VAZ pool size, responsive at long term); (ii) the kinetics of the VDE and ZE enzymes that determine the speed for xanthophyll conversion. They depend on post‐translational regulation of the enzymes and importantly, illumination conditions of the previous minutes, that is, light inhibits ZE‐ have a key effect at short term minutes (Küster et al. 2023); (iii) the amount of the VDE and ZE enzymes can also determine the speed of xanthophyll conversion. It is a less studied factor but the few available works with mutants indicate that the ratio VDE/ZE may be very relevant, and the natural regulators of this ratio are still unknown (Jahns and Bethmann 2025; Küster et al. 2023). Other processes such as the hydroxylation of β‐carotene can contribute to enhance Z formation (Davison et al. 2002) and this actually represents the pathway for increasing the size of the total VAZ pool.

The extent of quenching depends on how long ago and how much light a leaf has been exposed to (Matuszynska et al. 2016). The occurrence of sudden periods of strong illumination typically results in a rapid increase in AZ/VAZ, which is slowly reversible under low light, while the VAZ pool size is regulated over the long term, as part of the acclimation of leaves to their light environment (Niinemets et al. 2003). In fact, the VAZ pool size correlates linearly with the total dose of light energy received by a leaf (Esteban et al. 2015). The VAZ pool size responds plastically to changes in light, but its response, implying a profound reorganization of the photosynthetic apparatus, takes days or weeks (Niinemets et al. 2003).

Concepts such as plant memory, intelligence, behavior, communication, or even consciousness have been the subject of intense debates among the community of plant researchers (Trewavas et al. 2020; Calvo et al. 2020; Mallatt et al. 2021; Aphalo and Sandras 2022; Karban 2008). Among these terms, the existence of a kind of plant stress memory, also known as stress priming, is generally accepted. This memory can be based on different mechanisms: electrical signals in *Dionea* (Calvo et al. 2020), epigenetic changes (Molinier et al. 2006; Müller‐Xing et al. 2014) that can be transmitted to the next generation (Gallusci et al. 2023), gene expression (Auge et al. 2023), hormone action (Fleta‐Soriano and Munné‐Bosch 2015), and metabolites (Pinto‐Marijuan et al. 2017). Applying the classical definition of “plant memory” by Trewavas (2003) as the storage of information from previous events and the ability to retrieve it at a much later time influencing the response to current signals, the dynamics of the VAZ cycle and its involvement in NPQ regulation could be easily classified as a memory mechanism in plants. This concept has been repeatedly postulated by several authors who have proposed that protective mechanisms activated in response to high‐light stress represent a form of light memory in plants (Horton et al. 2005; Jahns and Holzwarth 2012). In this conceptual framework, the thylakoid, with its light‐harvesting systems, electron transport chain, and proton pumping mechanisms, would act as the sensor of the photon dose received by a leaf. Photon sensing, translated into a proton gradient, controls the formation of A + Z through the enzymes VDE and ZE. In such a mechanistic view, two opposing enzymatic processes contribute to the actual AZ/VAZ (Jahns and Bethmann 2025): on the one hand, the computation of light dose received during previous hours translated into VDE activity (memory formation), and on the other hand, the time‐related relaxation of AZ/VAZ caused by ZE activity (memory vanishing). Thus, the extent of quenching depends on how long ago and how much light a leaf has been exposed to (Matuszynska et al. 2016). These xanthophylls, which represent a kind of short‐lived storage form of light memory, ultimately determine the photoprotective and photosynthetic responses to current illumination. Furthermore, if we assume that A + Z is a form of memory storage, the long‐term regulation of the VAZ pool would represent another memory mechanism (Esteban et al. 2015).

In this study, we investigated, under natural lighting conditions, the relationship between AZ/VAZ at a given time and the cumulative light dose, defined as the product of light intensity and exposure duration. In particular, considering xanthophylls as components of light memory, the question to be addressed is how long this memory lasts. We consider two possible scenarios. In the first, the immediate light regime is the primary determinant of xanthophyll composition, and the strength of the relationship between cumulative light dose and AZ/VAZ decreases with time. In the second scenario, light memory strengthens over time, such that the strongest correlation is obtained when AZ/VAZ is related to the cumulative radiation dose integrated over a longer time window.

## 2 Materials and Methods

### 2.1 Experimental Design

Field experiments were performed in the Hyytiälä Field Station (Finland), Lat N 61°50′, Long E 24° 17′. The site has a boreal climate, with mean monthly temperatures ranging between −6°C in winter and 17°C in summer and annual rainfall around 700 mm. The forest is dominated by two conifers: spruce ( *Picea abies* ) and Scots pine ( *Pinus sylvestris* ), and its continuity is broken with abundant bogs and lakes.

Field measurements were performed in summer during two campaigns in July 2017 and June 2022 (Figure 1). A total of 17 species were studied, covering the main functional groups of the boreal forest: two lichens ( *Peltigera aphthosa* and *Cetraria islandica* ), two mosses ( *Polytrichum juniperinum* and *Sphagnum* sp.), three ferns and fern allies ( *Equisetum sylvaticum* , *Gymnocarpium dryopteris* , and *Lycopodium clavatum* ), two conifers ( *Pinus sylvestris* and *Picea abies* ), three herbaceous angiosperms ( *Alchemilla monticola* , *Convalaria majalis*, and *Maianthemum bifolium* ), three woody deciduous angiosperms ( *Betula pendula* , *Sorbus aucuparia* , and *Vaccinium myrtilus*), and two woody evergreen angiosperms ( *Vaccinium vitis‐idaea* and *Rhododendron tomentosum* ).

**FIGURE 1:**
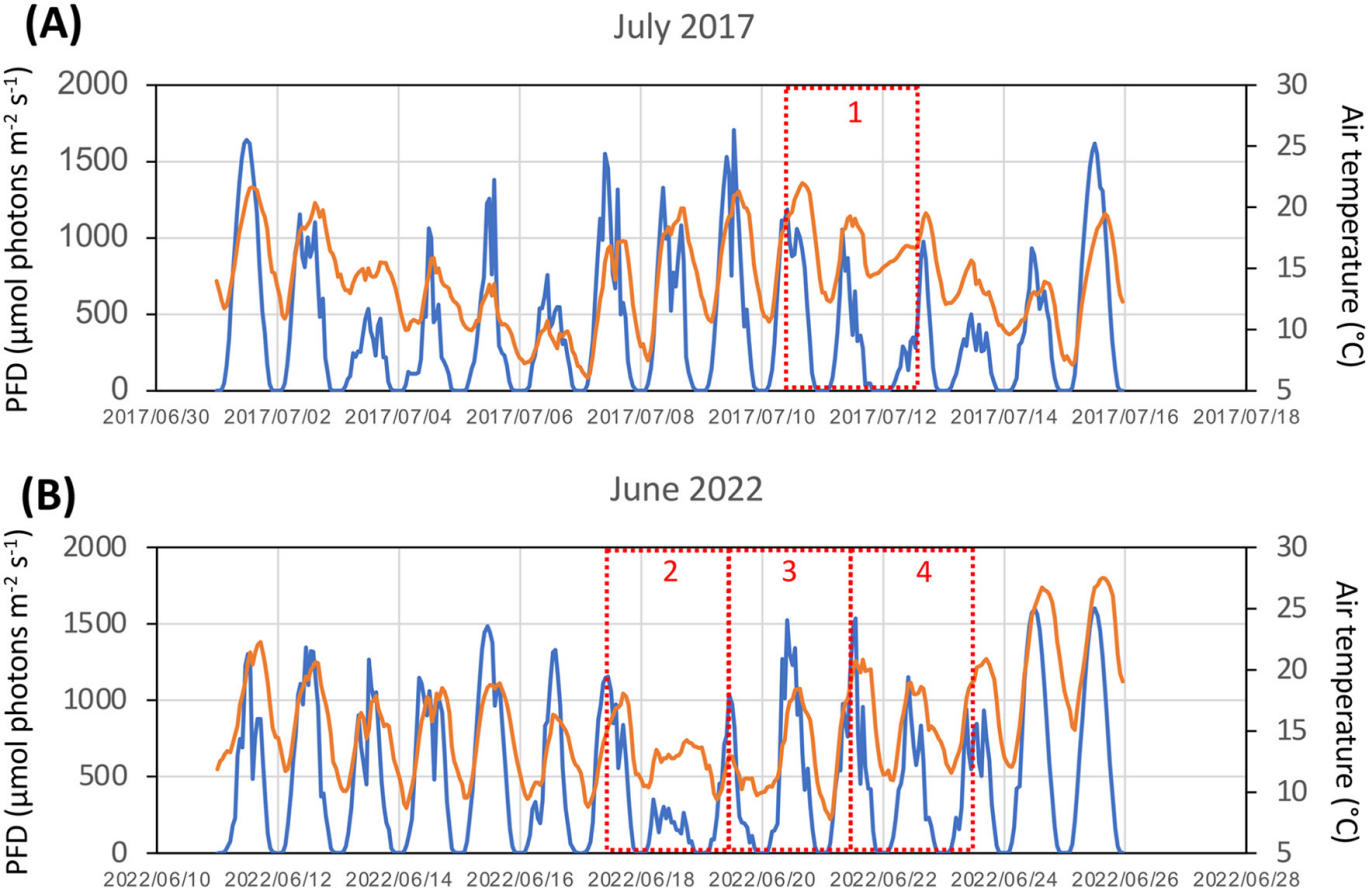
Meteorological conditions (photosynthetic photon flux density—PFD and temperature) at the SMEAR meteorological station in Hyytiälä during the field campaigns of 2017 (A) and 2022 (B). Dashed boxes indicate the 48 h periods of sample collection: Period 1 (*P. aphthosa, Sphagnum* sp., *E. sylvaticum* , *P. sylvestris* , *B. pendula* , *C. majalis* and *V. vitis‐idaea* ); period 2 ( *S. aucuparia* , *M. bifolium* , *A. monticola* and *G. dryopteris* ); period 3 ( *P. juniperinum* , *V. myrtillus* , *L. clavatum* ) and period 4 ( *R. tomentosum* , *P. abies* , *C. islandica* ). Photon dose in the open ground received during each period was 27.8 mol m^−2^ day^−1^ for Period 1; 24.9 mol m^−2^ day^−1^ for Period 2; 39.3 mol m^−2^ day^−1^ for Period 3 and 34.8 mol m^−2^ day^−1^ for Period 4. Blue line represents PFD and red line represents temperature.

Four “individuals” of each species, or cushions for the case of mosses, were monitored every 6 h during two consecutive days, starting and ending at midday, giving a total of nine time points. Thus, three consecutive middays and two midnights were studied for each species. Sampling days were planned based on the forecasted cloudiness, so that during the monitoring period the plants could experience a combination of both cloudy and sunny events (Figure 1). Once collected in the field, plant samples were immediately frozen in liquid nitrogen, stored at −80°C in the Field Station and transported within a dry shipper to the laboratory of the University of the Basque Country where they were stored at −80°C until pigment analysis. Through chlorophyll fluorescence measurements, maximal and actual efficiency of PSII were estimated at the same times of sampling (see below).

### 2.2 Estimation of Cumulative Photon Dose

To determine the Cumulative Photon Dose (CumPFD) received by each leaf/thallus over a certain time frame we used the Photon Flux Densities (PFD) information provided by radiometers (in μmol photons m^−2^ s^−1^) from a nearby meteorological station (Hyytiälä SMEAR 2). Values of CumPFD were corrected for each leaf by the fractions of diffuse and direct light reaching the leaves. These fractions were calculated with Gap Light Analyzer (GLA) Version 2.0 software using hemispherical photographs taken above the leaves with a Nikon Coolpix 4500 digital camera equipped with a Fisheye Converter FC‐E8. The proportion of monthly radiation below the canopy, relative to that in the open (Relative Irradiance, RI), was calculated for each canopy position. Actual CumPFD values, expressed in mol m^−2^, were calculated for the time intervals from 1 h to 24 h prior to sample collection (CumPFD_1_, CumPFD_2_, …, CumPFD_24_). CumPFDs ranged between 0 and 3.81 mol m^−2^ for CumPFD_1_ and between 2.35 and 36.22 mol m^−2^ for CumPFD_24_.

### 2.3 Fluorescence Measurements

The actual efficiency of PSII (Φ_PSII_) was measured on the same leaves collected for pigment analysis by using a portable modulated Plant Stress Kit Fluorometer (Opti‐Sciences). In leaves exposed to natural solar radiation, the operating quantum efficiency of PSII was estimated as Φ_PSII_ = (*F*_m_′ − *F*_s_)/*F*_m_′, where *F*_m_′ is the maximum Chl*a* fluorescence induced with a saturating pulse, and *F*_s_ is the actual Chl*a* fluorescence under illumination. Saturation pulses to calculate *F*_m_′ values were applied as described by Loriaux et al. (2013). Photon flux density (PFD) was measured using the Leaf‐Clip built‐in irradiance sensor.

In a parallel set of dark‐adapted leaves (30 min), the maximum Chl*a* fluorescence (*F*_m_) was induced with a saturating pulse (0.8 s duration, 7000 μmol m^−2^ s^−1^ PFD), while the initial fluorescence (*F*_o_) was recorded using red modulated measuring light (< 1 μmol photons m^−2^ s^−1^). The maximal efficiency of PSII (*F*_v_/*F*_m_) was estimated using the ratio *F*_v_/*F*_m_ = (*F*_m_ − *F*_o_)/*F*_m_. This methodological approach did not allow the estimation of NPQ (Zuo 2025) as both *F*_m_ and *F*_m_′ were obtained using different devices and protocols and consequently are not comparable.

### 2.4 Pigment Analyses

Samples for pigment analysis were extracted with pure acetone buffered with 0.5 g CaCO_3_ L^−1^. The plant material was ground under liquid N_2_, and the extracts were centrifuged at 16,100 × *g* and 4C for 10 min. The pellet was then re‐suspended in pure acetone, mixed in a vortex, and centrifuged again at 16,100 × *g* and 4°C for 10 min. Both supernatants were pooled and filtered through a 0.2 μm polytetrafluoroethylene filter and then injected into the HPLC system. The pigment composition (chlorophyll *a*; chlorophyll *b*, neoxanthin, violaxanthin, antheraxanthin, lutein, zeaxanthin, and β‐carotene) was analysed by HPLC using a reverse phase C18 column (Waters) as described previously (García‐Plazaola and Becerril 1999; Fernández‐Marín et al. 2018) using a photodiode array detector (Waters model 996).

From the analyses, three ratios were derived and considered as potential mechanisms of memory storage:

- Chlorophyll *a*/*b* (Chl *a*/*b*) was calculated as the molar ratio between chlorophyll *a* and *b*. Since all Chl *b* is located in the light harvesting complexes (LHCs) while Chl *a* can be present both in the antennae and core complexes (CCs), Chl *a*/*b* is a good indicator of the antenna size (Esteban et al. 2015), which is dynamically regulated by long‐term changes in irradiance. This ratio can also be affected by the stoichiometry of PSI to PSII, as most of the Chl *b* pool is bound to LHCII.
- Xanthophyll cycle pool (VAZ) per chlorophyll (VAZ/Chl): The total VAZ pool was calculated as the sum of violaxanthin (V), antheraxanthin (A), and zeaxanthin (Z) and expressed on a chlorophyll (*a* + *b*) basis. As a consequence, the size of the VAZ pool is usually proportional to the light environment where the plant or tissue develops; it is highly responsive to light fluctuations and determines the potential to form new Z molecules upon illumination (Esteban et al. 2015).
- De‐epoxidation index (AZ/VAZ): AZ/VAZ was calculated as the sum of A and Z divided by the sum of V, A, and Z. It represents the performance state of the VAZ cycle, which is proportional to light excess and/or to the extent of other stress factors (Demmig‐Adams and Adams 1996; Fernández‐Marín et al. 2021). This parameter modulates the development of NPQ and is usually proportional to it.

### 2.5 Statistical Analysis

Pearson's product–moment correlation coefficient (*r*) was used to assess linear relationships among variables. Specifically, the strength of linear correlations between cumulative photon doses (CumPFDs) and pigment indices was tested across time intervals from 1 to 24 h preceding sample collection. Statistical significance was evaluated using two‐tailed tests, and associated *p* values were calculated.

## 3 Results

With the purpose of verifying whether chloroplast pigments can act as biochemical mechanisms of memory storage, time‐related changes in pigment composition were studied along 48 h in 17 species representatives of the main functional groups of the boreal forest in Southern Finland. All species were collected in the understory of a relatively open mixed spruce‐pine forest (average canopy transparency 48% ± 13%, relative irradiance in Table 1). Lichens ( *P. aphtosa* and *C. islandica* ) showed the lowest chlorophyll content among all the groups, on a dry weight basis, being 20–30 fold lower than in herbaceous angiosperms. On the contrary, Lutein (L) content was much higher in lichens than in any other group. Relative irradiance correlated positively with VAZ/Chl (*p* < 0.01) and marginally correlated (*p* = 0.1) with Chl*a*/*b*, but it did not show any relationship with the average AZ/VAZ (data not shown).

**TABLE 1:** Average pigment composition of the studied species.

Species	RI	Chl *a* + *b*	Chla/b	N/Chl	L/Chl	VAZ/Chl	β‐C/Chl	AZ/VAZ
*P. aphthosa*	37	0.28 ± 0.04	3.09 ± 0.04	50.0 ± 0.66	200.0 ± 2.7	84.7 ± 3.1	52.3 ± 1.6	0.478 ± 0.081
*C. islandica*	48	0.21 ± 0.03	3.14 ± 0.25	67.7 ± 5.3	277.0 ± 25.1	88.8 ± 6.8	90.7 ± 9.3	0.651 ± 0.073
*P. juniperinum*	70	1.80 ± 0.11	3.12 ± 0.03	38.3 ± 0.6	134.5 ± 1.1	83.8 ± 1.4	88.5 ± 0.7	0.367 ± 0.046
*Sphagnum* sp.	37	1.46 ± 0.12	2.51 ± 0.02	46.3 ± 1.1	160.0 ± 1.9	54.1 ± 2.0	72.7 ± 1.1	0.427 ± 0.056
*E. sylvaticum*	20	4.08 ± 0.51	2.87 ± 0.01	38.6 ± 0.3	110.6 ± 0.8	42.8 ± 0.6	53.7 ± 0.6	0.215 ± 0.011
*G. dryopteris*	52	4.15 ± 0.19	3.20 ± 0.02	41.5 ± 18.4	121.3 ± 1.0	108.5 ± 4.1	79.5 ± 0.7	0.346 ± 0.080
*L. clavatum*	39	1.96 ± 0.22	3.34 ± 0.02	38.5 ± 0.5	139.3 ± 1.3	65.9 ± 1.1	78.1 ± 0.6	0.301 ± 0.040
*P. sylvestris*	51	1.14 ± 0.06	2.37 ± 0.06	46.9 ± 1.1	154.1 ± 3.6	28.2 ± 1.4	92.2 ± 2.1	0.338 ± 0.042
*P. abies*	49	1.14 ± 0.03	3.32 ± 0.03	41.9 ± 0.8	167.4 ± 1.9	72.8 ± 2.1	73.9 ± 1.2	0.277 ± 0.060
*A. monticola*	49	6.01 ± 0.21	3.44 ± 0.01	32.6 ± 1.4	109.9 ± 0.9	90.5 ± 0.6	99.8 ± 0.6	0.246 ± 0.065
*C. majalis*	36	4.51 ± 0.29	3.19 ± 0.04	44.2 ± 0.6	154.1 ± 2.9	58.1 ± 2.1	83.2 ± 2.7	0.428 ± 0.066
*M. bifolium*	66	5.19 ± 0.27	3.33 ± 0.02	38.1 ± 1.4	121.4 ± 1.3	96.6 ± 4.0	78.9 ± 0.6	0.467 ± 0.074
*B. pendula*	49	4.35 ± 0.19	3.39 ± 0.02	35.3 ± 0.4	113.7 ± 1.2	58.1 ± 1.8	89.2 ± 1.1	0.273 ± 0.032
*S. aucuparia*	60	3.10 ± 0.09	3.68 ± 0.02	38.5 ± 0.4	126.1 ± 1.1	146.4 ± 2.9	93.4 ± 0.7	0.312 ± 0.087
*V. myrtillus*	70	3.78 ± 0.18	3.34 ± 0.04	34.1 ± 0.5	128.5 ± 1.5	102.0 ± 5.7	99.7 ± 1.0	0.380 ± 0.055
*V. vitis‐idaea*	37	1.49 ± 0.08	2.67 ± 0.09	37.3 ± 0.9	150.4 ± 3.9	41.0 ± 2.4	117.3 ± 3.1	0.519 ± 0.033
*R. tomentosum*	41	2.22 ± 0.08	3.12 ± 0.02	38.2 ± 0.5	123.1 ± 1.0	58.6 ± 1.1	87.8 ± 0.9	0.308 ± 0.033

*Note:* Data are the average of all measurements of each species (*n* = 36). RI represents the average relative irradiance (%) at the site of sampling of each species. Total chlorophyll (Chl *a* + *b*) is expressed in μmol Chl g^−1^DW, Chl*a*/*b* is expressed in mol mol^−1^, neoxanthin (N), lutein (L), β‐carotene (β‐C) and violaxanthin + antheraxanthin + zeaxanthin (VAZ) to Chl ratios are expressed in mmol mol^−1^ (antheraxanthin + zeaxanthin)/VAZ ratio is expressed in mol mol^−1^.

When all species, functional groups and sampling points were included, the ratio AZ/VAZ showed a strong negative correlation with both the actual quantum yield (Φ_PSII_) and with the maximum quantum yield (*F*_v_/*F*_m_) (Figure 2) supporting the existence of a mechanistic relationship between the operation of xanthophyll cycle and the photochemical activity. The stronger correlation observed for Φ_PSII_ compared with *F*_v_/*F*_m_ is likely because of the fact that pigment analyses and Φ_PSII_ were performed on the same leaves. With a few exceptions, notably lichens, both correlations were also verified for each functional group when analysed separately (Table S1). The other two parameters studied as potential mechanisms of light memory storage (VAZ/Chl and Chl*a*/*b*) were unrelated to photochemical activity (*F*_v_/*F*_m_ and Φ_PSII_; data not shown). Both pigment ratios were strongly correlated with each other (Figure 2) and this correlation also occurred when each group was considered separately, except for the case of lichens (Table S1). Nevertheless, this correlation was at least partially artefactual and caused by the constitutive differences among functional groups (e.g., Chl*a*/*b* was never lower than 3.2 in woody deciduous angiosperms, and never higher than 2.8 in conifers).

**FIGURE 2:**
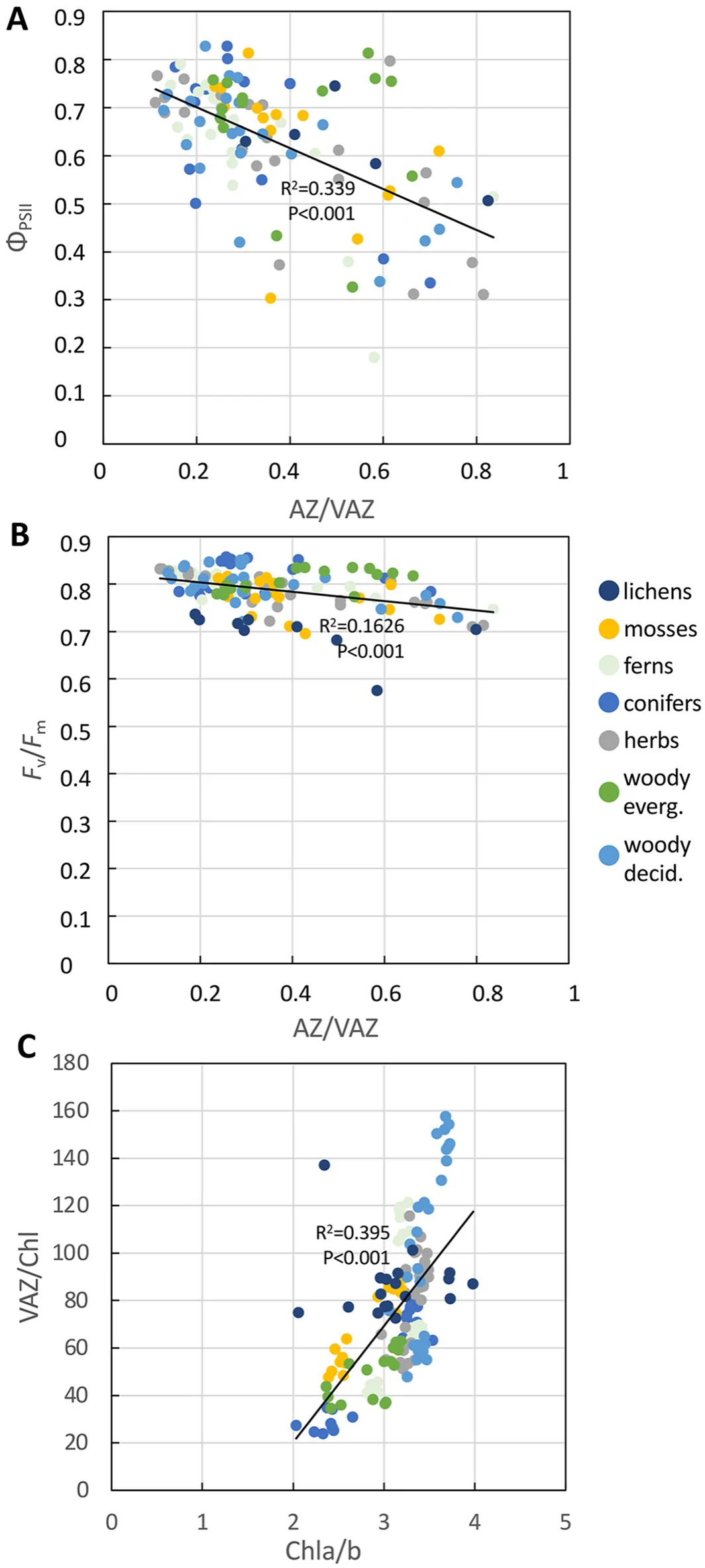
Relationships between AZ/VAZ and photochemical parameters (maximal and actual quantum yield of PSII, *F*_v_/*F*_m_ (A) and Φ_PSII_ respectively) and relationship between VAZ/Chl and Chl*a*/*b* (C) for all species and time points. (B) *F*_v_/*F*_m_ and Φ_PSII_ for lichens were included only when fluorescence was not quenched by desiccation.

The strength of the correlations between AZ/VAZ, the proposed mechanism of memory storage, and the light dose (CumPFD) received by a plant over a certain cumulative time window from 1 to 24 h is shown in Figure 3. Overall, this figure highlights the illumination interval that best explains the AZ/VAZ, or in other words the timespan of light memory. For the case of lichens, the two species studied differed greatly in the pattern of time response of the maximal correlation coefficient of AZ/VAZ vs. CumPFD, with *C. islandica* being significant between 3 and 12 h, and *P. aphthosa* between 21 and 24 h. Ferns and mosses showed remarkably similar patterns, being significant during the first 5–10 h peaking between 2 and 5 h. The only exception was *L. clavatum* that did not show any significant correlation during the 24 h of time interval studied. The two conifer species studied showed remarkably similar responses, with significant correlations between AZ/VAZ and CumPFD from 8 to 15 h and reaching a maximum at 12 or 14 h. In contrast, among the angiosperms studied, the response was more diverse. Thus, in woody deciduous species the response pattern was similar to that observed in mosses and ferns (strongest correlations at short time intervals). One of these species, *V. myrtillus* , described the same pattern, but was only marginally significant (*p* < 0.1). By contrast, evergreen woody angiosperms exhibited a pattern with much longer (> 8 h) intervals of significance (which greatly resembled those observed in conifers). Finally, the herbaceous angiosperms studied here differed among them with one species ( *M. bifolium* ) resembling the short‐term response of mosses, ferns and deciduous angiosperms, whereas two others ( *C. majalis* and *A. monticola* ) peaked at longer times. Interestingly, for some species and time intervals, the correlation between cumulative light dose and AZ/VAZ was very strong (*R* ^2^ > 0.8; *p* < 0.001), this was the case for the short‐term intervals (1–4 h) in *Sphagnum* sp., *G. dryopteris* and *S. aucuparia* and for longer terms (8–16 h) in *C. majalis* and *A. monticola* .

**FIGURE 3:**
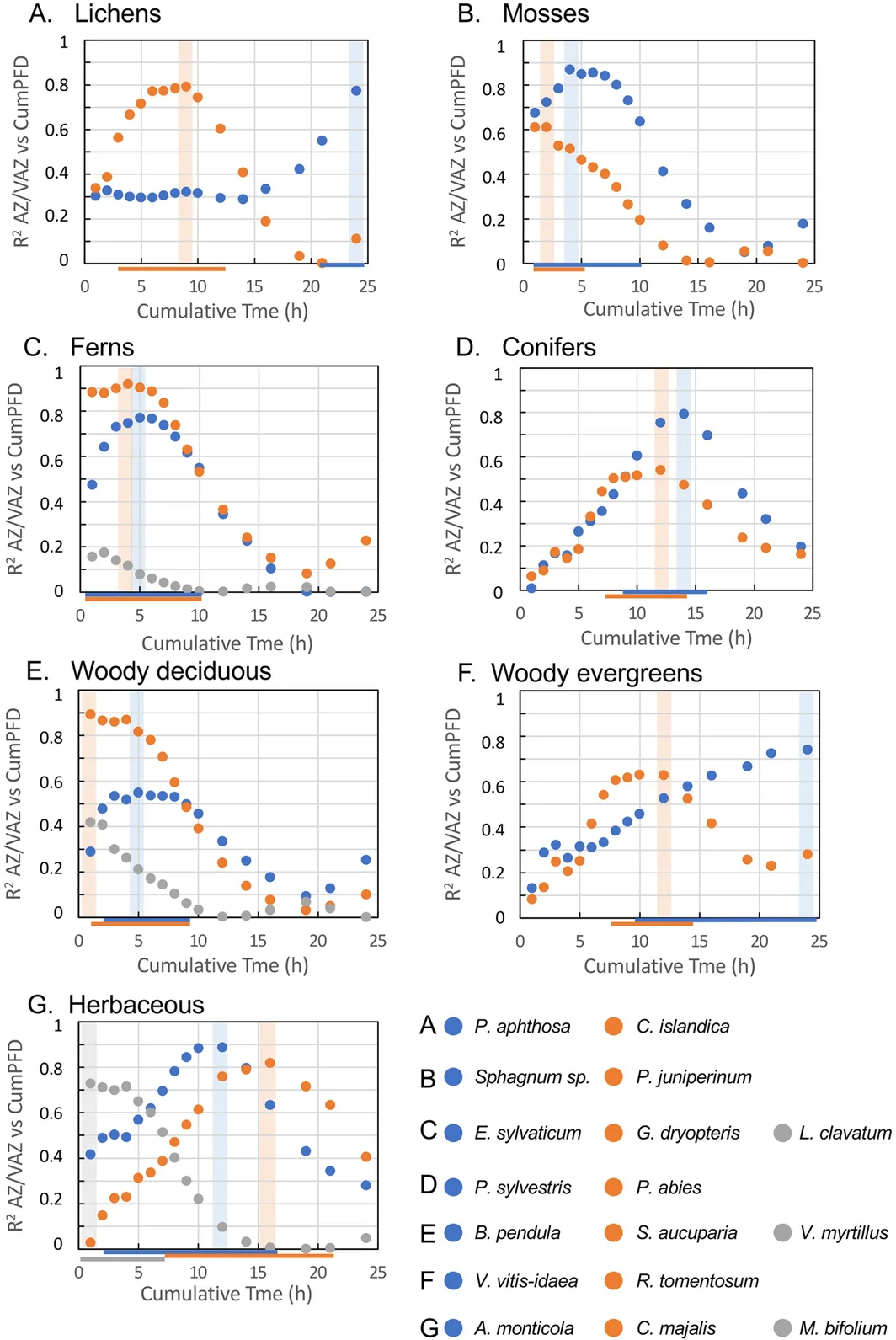
Pearson correlation coefficients (*R* ^2^) of the relationship between AZ/VAZ and the cumulative photon dose (CumPFD) previously received by the leaf during a certain time interval (from 1 to 24 h, CumPFD_1_ to CumPFD_24_). Horizontal bars at the base of each panel denote significant correlations at *p* < 0.05, and shaded background indicates the cumulative time at which CumPFD best correlates with AZ/VAZ. Data for AZ/VAZ and CumPFD for the time windows of greatest significance in each plot are reported in Table S2.

The cumulative time‐dependent strength of the linear correlations between Φ_PSII_ and the light dose (CumPFD) was also tested (Figures 4 and 5). In general, these response patterns were quite similar to those observed for the relationship between AZ/VAZ and CumPFD (Figure 3). There were apparently two major exceptions to this general rule, the lichen *P. aphthosa* and the evergreen shrub *R. vitis‐idaea*. In both species, the cumulative light dose that best correlated with Φ_PSII_ was much shorter than that corresponding to the strongest correlation with AZ/VAZ. As a result of the parallel response patterns, these two parameters AZ/VAZ and Φ_PSII_ were significantly correlated for most species (Figures 4 and 5, right panels), with the exception of *C. islandica* , *P. juniperinum* , *E. sylvaticum* , *G. dryopteris* , and *B. pendula* . Interestingly, all of these species, except *B. pendula* , are cryptogams.

**FIGURE 4:**
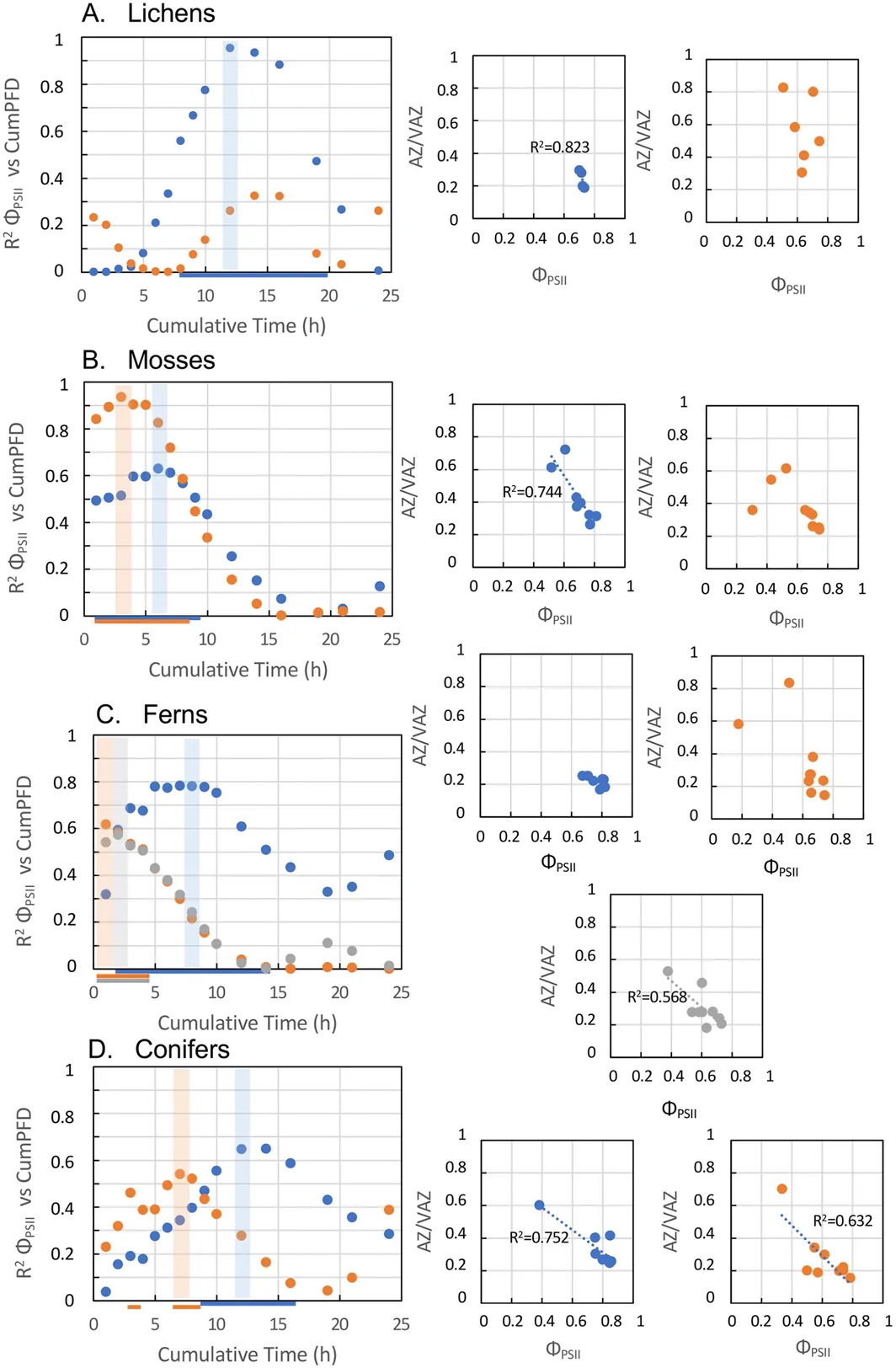
Pearson correlation coefficients (*R* ^2^) of the relationship between quantum yield of PSII (Φ_PSII_) and the cumulative photon dose (CumPFD) previously received by the leaf during a certain time interval (from 1 to 24 h, CumPFD_1_ to CumPFD_24_) for lichens (A), mosses (B), ferns (C), and conifers (D). Horizontal bars at the base of each panel denote significant correlations at *p* < 0.05, shaded background indicates the cumulative time at which CumPFD best correlates with Φ_PSII_. Right panels show linear correlations between Φ_PSII_ and AZ/VAZ for each species. Whenever significant *R* ^2^ values and regression lines are shown. Color codes for species are the same as in Figure 3.

**FIGURE 5:**
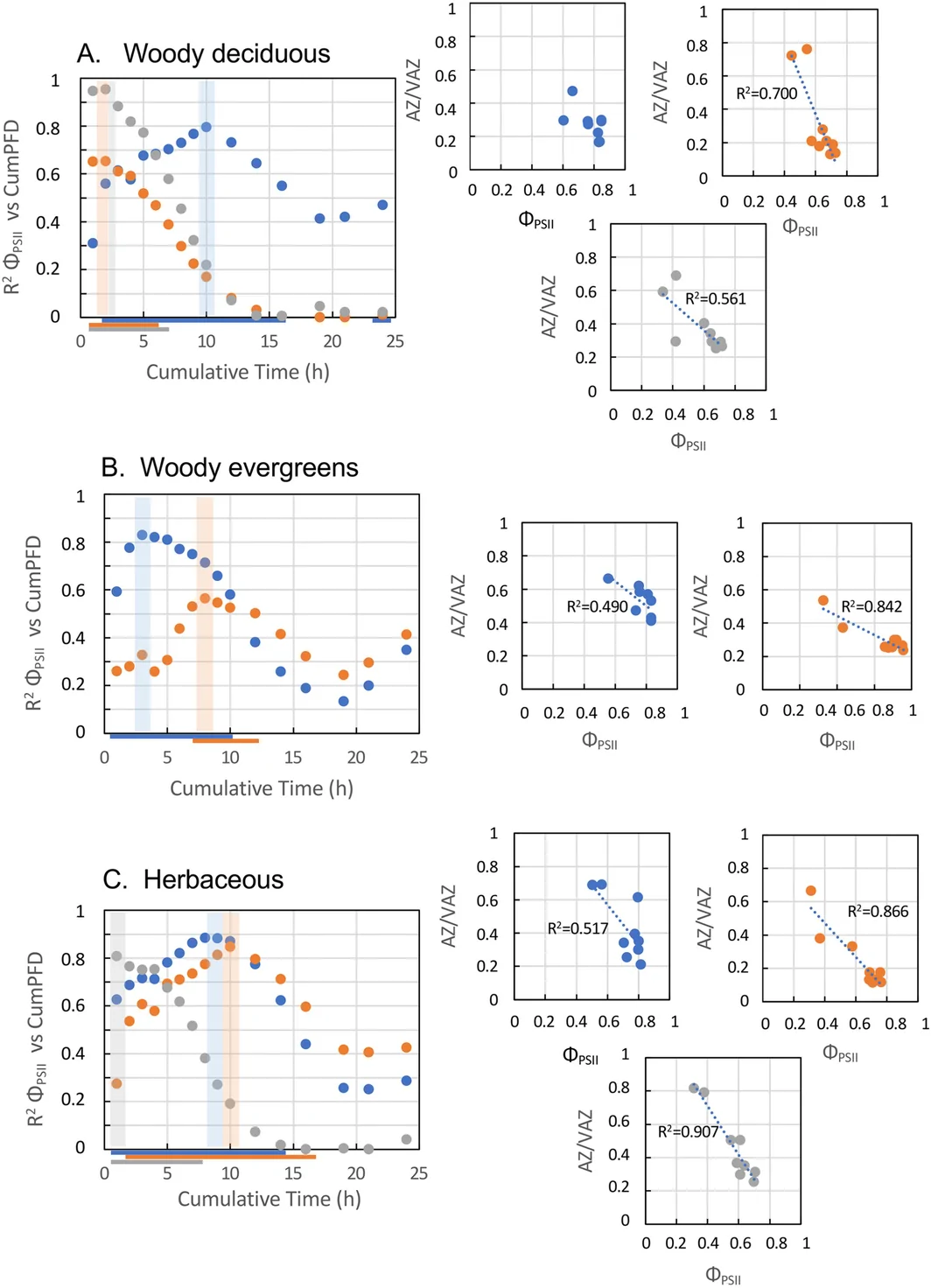
Left panels: Pearson correlation coefficients (*R* ^2^) of the relationship between quantum yield of PSII (Φ_PSII_) and the cumulative photon dose (CumPFD) previously received by the leaf during a certain time interval (from 1 to 24 h, CumPFD_1_ to CumPFD_24_) for woody deciduous (A), woody evergreen (B) and herbaceous angiosperms (C). Horizontal bars at the base of each panel denote significant correlations at *p* < 0.05, shaded background indicates the cumulative time at which CumPFD best correlates with Φ_PSII_. Right panels show linear correlations between Φ_PSII_ and AZ/VAZ for each species. Whenever significant *R* ^2^ values and regression lines are shown. Color codes for species are the same as in Figure 3.

## 4 Discussion

Present results indicate that for 15 of the 17 species studied, actual AZ/VAZ levels are more strongly influenced by the total amount of radiation received by a leaf over a certain time window (CumPFD) than by instantaneous light intensity. These strong correlations support the idea that photons are primarily sensed and counted by the photosynthetic system (Park et al. 1995, 1996). After basic computation, this quantitative information about the light history experienced by chloroplasts is translated into xanthophyll cycle activity, being written and subsequently stored as AZ/VAZ (Szechyńska‐Hebda et al. 2010). The time window that maximizes the correlation between AZ/VAZ and CumPFD differs among species, with two main patterns: species whose strongest correlation is attained with the cumulative light received over short periods (< 5 h), and species whose highest correlation is achieved for cumulative light received over longer periods (> 10 h). Beyond these maxima, the strength of the correlation progressively vanishes. This is not surprising under the frame of current models of xanthophyll cycle activity and interconversions that show the content of de‐epoxidised xanthophylls as the result of two opposed reactions of epoxidation and de‐epoxidation with different kinetics and requirements (Jahns and Bethmann 2025). Overall, these results support that photons are primarily sensed and counted by the photosynthetic system, and after basic computation, the cumulative light information is written and stored as AZ/VAZ values.

Importantly, CumPFD is not the only environmental determinant of AZ/VAZ. Thus, given that the xanthophyll cycle is in essence an enzymatic process, its kinetics are temperature‐dependent (Bilger and Björkman 1991) and temperatures below a certain threshold could also impair VAZ cycle operation (Demmig‐Adams et al. 1989). However, temperatures during the field experiments and previous days' temperatures were moderate and the thermal range narrow, between 10.6°C and 21.6°C in 2017 and 7.8°C and 20.0°C in 2022, making a dominant effect of temperature on the VAZ cycle performance less likely. To test this possibility, we compared the strength of linear correlations between AZ/VAZ and the mean temperature over the preceding time interval (Tmean) with that of the relationship between AZ/VAZ and CumPFD (Table S4). Although significant correlations with Tmean were observed in some species and time intervals, correlations with CumPFD were consistently stronger across all species, suggesting that variations of AZ/VAZ are more closely associated with irradiance.

Given the substantial body of correlative and mechanistic evidence supporting that AZ/VAZ is strongly related to the photosynthetic efficiency (Kromdijk et al. 2016), the relationship between AZ/VAZ and CumPFD provides support for the existence of such form of light memory that persists over time. The involvement of xanthophylls as mechanisms of stress memory has been previously postulated (Horton and Ruban 2004; García‐Plazaola et al. 2012; Pinto‐Marijuan et al. 2017), but here we provide quantitative evidence of the timescales required for its maximum activation. Furthermore, to fulfill the concept of memory, the information stored should be retrievable at a later time producing a physiological effect (Trewavas 2003). This is confirmed in Figures 2, 4, and 5 which show that AZ/VAZ correlates with basic indicative parameters of photochemical activity of PSII (*F*_v_/*F*_m_ and Φ_PSII_) in most species. At this regard, the weaker correlation between *F*_v_/*F*_m_ and AZ/VAZ, compared with that of Φ_PSII_ and AZ/VAZ (Figure 2), is that the samples used for pigment analyses were collected in illuminated leaves, whereas determination of *F*_v_/*F*_m_ requires 30 min of dark acclimation. Although this could contribute, the strength of the correlation does not improve (*r* ^2^ = 0.169, *p* < 0,015) when only samples collected at midnight are considered across all species.

With the limitation arisen from our non‐manipulative design (i.e., light conditions during each sampling period differed by a factor of 1.4), from present data, we can propose the existence of two major groups/strategies regarding the involvement of xanthophylls de‐epoxidation state in light memory: plants with short‐term memory, usually characterized by soft leaves like mosses, ferns and deciduous trees, and plants with longer‐term memory and usually tough leaves (most of them evergreen species). While the first one would respond more accurately to oscillations in the light regime, the second one would tend to attenuate the effect of environmental fluctuations. The requirement of a certain period of light accumulation to achieve maximum values represents a conservative strategy and agrees with the fact that species requiring longer time periods to reach the highest correlation with AZ/VAZ are also those with longer leaf lifespans (evergreens). However, this “legacy effect” could also have a detrimental effect eventually preventing the optimum photosynthetic utilization of favorable environmental periods (Kromdijk et al. 2016). In addition to this, poikilohydric organisms, such as lichens and mosses, represent a distinct case in which the de‐epoxidation state may be primarily determined by water availability rather than by light conditions (Fernández‐Marín et al. 2010, 2013, 2021). Indeed, this correlation was observed during the experiment (data not shown) where the *F*_v_/*F*_m_ values increased during night when hydrated and decreased during midday when the species where completely dry. These observations agree with increasing evidence of dehydration‐mediated of AZ/VAZ accumulation in desiccation‐tolerant organisms (Fernández‐Marín et al. 2021).

Two other pigment ratios indicative of photosynthetic performance and strongly modulated by light environment: Chl*a*/*b* and VAZ/Chl (Esteban et al. 2015) were also examined in the frame of the present study. The first is an indicator of the structure of the photosynthetic apparatus (Chl*a* is present in core complexes and light‐harvesting antennae, while Chl*b* is bound only to antennae proteins) and scales positively with irradiance (Esteban et al. 2015). The second also correlates with irradiance and, in principle, the larger the VAZ pool is, the higher is the potential to form A + Z. Consequently, this light‐induced upregulation of the VAZ pool size can also be considered as part of the plant memory (Demmig‐Adams et al. 2022). Nevertheless, we did not find any consistent pattern when calculating time windows shorter than 24 h.

## 5 Concluding Remarks

Plants, as sessile organisms, base most of their fitness on the ability to respond to environmental fluctuations. In a manner reminiscent of the closing quotation of Blade Runner: “All those moments will be lost in time, like tears in rain”, the de‐epoxidation state‐based plant memory is likewise ephemeral, vanishing with time and rarely persisting beyond 24 h. In fact, considering that this type of memory represents the ability to calculate and remember CumPFD over periods of time and make later photosynthetic “decisions,” it could be particularly relevant for cultivated species. Thus, in terms of productivity, a desirable target could be to produce “amnesic” plants that lack memory of a specific type of stress or alternatively, “traumatized” plants in which the experience of a past stress period never vanishes.

## Author Contributions

J.I.G.‐P. and B.F.‐M. conceived the idea. All authors conducted the field measurements and analyses. M.I.A. and B.F.‐M. did a preliminary data analysis. J.I.G.‐P. and B.F.‐M. drafted the manuscript. All authors contributed to the final version of the manuscript.

## Funding

This work was supported by European Union's Horizon 2020 Project INTERACT (730938), MCIN/AEI, ERDF (PID2022‐139455NB‐C32), MCIN/AEI, European Union Next‐GenerationEU/PRTR (RYC2021‐031321‐I), MICIU/AEI, FSE+ (JDC2024‐053975‐I), and Basque Government (IT1648‐22).

## Supporting information


**Table S1:** Pearson correlation coefficients (*R* ^2^) of the relationship between the maximal and actual quantum yield of PSII (Fv/Fm and ΦPSII) and AZ/VAZ and of the relationship between VAZ/Chl and Chla/b for all species. Asterisks denote statistical significance at *p < 0.01; **p < 0.01 and ***p < 0.0001.
**Table S2:** Values of CumPFD and AZ/VAZ during the time windows showing the most statistically significant linear correlations between both parameters.
**Table S3:** original data set (AZ/VAZ, ΦPSII and Fv/Fm) at each of the nine different measuring time points (0–48 h).
**Table S4:** Highest value of Pearson correlation coefficients (*R* ^2^) of the relationship between AZ/VAZ and cumulative photon dose (CumPFD) previously received by the leaf during a certain time interval (from 1 to 24 h, CumPFD_1_ to CumPFD_24_) (first column), or the mean temperature (Tmean) over the same time intervals (third column). Second and fourth column represent the time interval at which the strongest correlations were observed.

## Data Availability

The data that support the findings of this study are available on request from the corresponding author.
