# Supplementary material for: Here Comes the Sun: Xanthophylls as Components of Plant Memory
Source: Physiol Plant. 2026 Oct 1;178(5):e71136. doi: 10.1111/ppl.71136 (PMC13630484; doi:10.1111/ppl.71136)
Supplement: Supplementary file 1 [file PPL-178-e71136-s001.pdf]

Supplementary Files

Here comes the sun: xanthophylls as components of Plant Memory

José Ignacio García-Plazaola[1*]; Miren Irati Arzac[1,3]; Águeda M. González-Rodríguez[2]; Laura Díaz-Jiménez[1,2]; Alicia V. Perera-Castro[4]; Beatriz Fernández-Marín[1]

Group	Fv/Fm *vs* AZ/VAZ	Φ_{PSII} *vs* AZ/VAZ	VAZ/Chl *vs* Chl*a*/*b*
Conifers	0 *n.s.*	0.356 **	0.892 ***
Ferns	0.402 ***	0.505 ***	0.264 **
Herbaceous	0.607 ***	0.481 ***	0.358 ***
Bryophytes	0.184 *n.s.*	0.298 *	0.909 ***
Woody deciduous	0.309 **	0.439 ***	0.563 ***
Woody evergreens	0.357 **	0.005 *n.s.*	0.462 **
Lichens	0.272 *n.s.*	0.343 *n.s.*	0.015 *n.s.*

Table S1: Pearson correlation coefficients (R^2) of the relationship between the maximal and actual quantum yield of PSII (Fv/Fm and ΦPSII) and AZ/VAZ and of the relationship between VAZ/Chl and Chl*a*/*b* for all species. Asterisks denote statistical significance at P<0.01 (*); P<0.01 (**) and P<0.001 (***).

Table S2

P. sylvestris	
$CumPFD_{14}$	AZ/VAZ
15.32	0.40
22.08	0.60
11.42	0.27
4.41	0.27
10.36	0.30
11.33	0.41
3.37	0.25
0.89	0.26
4.44	0.25

P. abies	
$CumPFD_{14}$	AZ/VAZ
12.63	0.34
19.16	0.70
9.76	0.22
2.41	0.19
9.78	0.20
13.99	0.20
6.05	0.15
1.42	0.20
8.02	0.30

C. majalis	
$CumPFD_{16}$	AZ/VAZ
10.82	0.69
16.27	0.69
11.03	0.61
4.57	0.25
7.32	0.34
8.73	0.40
3.53	0.30
1.01	0.21
3.13	0.35

A. monticola	
$CumPFD_{12}$	AZ/VAZ
13.41	0.38
19.93	0.67
9.22	0.17
2.46	0.17
3.86	0.13
4.27	0.13
2.14	0.12
1.04	0.11
8.82	0.33

M. bifolium	
$CumPFD_{1}$	AZ/VAZ
2.67	0.82
1.44	0.35
0.00	0.31
0.54	0.37
0.46	0.50
0.40	0.50
0.00	0.25
0.57	0.30
2.38	0.79

Sphagnum sp.	
$CumPFD_{5}$	AZ/VAZ
7.19	0.72
4.40	0.43
0.04	0.31
1.74	0.37
4.43	0.61
0.63	0.39
0.00	0.32
0.40	0.26
2.25	

P. juniperinum	
$CumPFD_{2}$	AZ/VAZ
5.29	0.61
0.90	0.34
0.00	0.25
0.85	0.26
6.70	0.36
2.40	0.36
0.00	0.24
2.45	0.33
6.82	0.55

E. sylvaticum	
$CumPFD_{5}$	$CumPFD_{5}$
3.89	
2.38	0.25
0.02	0.17
0.94	0.22
2.39	0.25
0.34	0.23
0.00	0.18
0.21	0.19
1.22	0.23

G.dryopteris	
$CumPFD_{4}$	AZ/VAZ
8.86	0.84
5.16	0.38
0.09	0.23
1.51	0.23
1.87	0.27
1.31	0.27
0.04	0.15
0.78	0.16
6.91	0.58

L. clavatum	
$CumPFD_{2}$	AZ/VAZ
2.95	0.28
0.50	0.20
0.00	0.18
0.48	0.28
3.73	0.28
1.34	0.24
0.00	0.45
1.36	0.28
3.80	0.28

S. aucuparia	
$CumPFD_1$	AZ/VAZ
2.43	0.76
1.31	0.28
0.00	0.19
0.49	0.18
0.42	0.21
0.36	0.21
0.00	0.14
0.52	0.13
2.16	0.72

V. myrtillus	
$CumPFD_1$	AZ/VAZ
2.52	0.69
0.42	0.34
0.00	0.26
0.48	0.25
3.21	0.29
1.36	0.29
0.00	0.29
1.42	0.40
3.81	0.59

B. pendula	
$CumPFD_5$	AZ/VAZ
9.53	0.47
5.83	0.29
0.05	0.22
2.31	0.27
5.86	0.29
0.83	0.30
0.00	0.17
0.53	0.29
2.98	0.16

V. vitis-idaea	
$CumPFD_{24}$	AZ/VAZ
16.42	0.66
17.00	0.62
16.97	0.57
16.09	0.58
13.37	0.47
9.17	0.53
9.14	0.43
7.79	0.41
4.37	0.41

R. tomentosum	
$CumPFD_{10}$	AZ/VAZ
10.55	0.37
13.73	0.53
4.56	0.25
1.20	0.26
8.17	0.30
10.21	0.30
3.20	0.24
0.82	0.27
6.70	0.25

C. islandica	
$CumPFD_9$	AZ/VAZ
12.25	0.80
14.56	0.80
4.46	0.58
1.35	0.50
9.49	0.82
10.30	0.82
2.29	0.41
0.93	0.31
7.79	0.82

P. aphthosa	
$CumPFD_{24}$	AZ/VAZ
16.42	0.66
17.00	0.59
16.97	0.67
16.09	0.72
13.37	0.69
9.17	0.30
9.14	0.20
7.79	0.19
4.37	0.28

Table S2: Values of CumPFD and AZ/VAZ during the time windows showing the most statistically significant linear correlations between both parameters.

Species	Time point	AZ/VAZ	Fv/Fm	Φ_{PSII}
P. sylvestris	**1** 12:00	0.40	0.83	0.75
P. sylvestris	**2** 18:00	0.60	0.81	0.38
P. sylvestris	**3** 24:00	0.27	0.84	0.83
P. sylvestris	**4** 6:00	0.27	0.85	0.80
P. sylvestris	**5** 12:00	0.30	0.85	0.75
P. sylvestris	**6** 18:00	0.41	0.85	0.85
P. sylvestris	**7** 24:00	0.25	0.85	0.85
P. sylvestris	**8** 6:00	0.26	0.86	0.86
P. sylvestris	**9** 12:00	0.25	0.85	0.85
P. abies	**1** 12:00	0.34	0.78	0.55
P. abies	**2** 18:00	0.70	0.78	0.33
P. abies	**3** 24:00	0.22	0.79	0.74
P. abies	**4** 6:00	0.19	0.79	0.57
P. abies	**5** 12:00	0.20	0.77	0.50
P. abies	**6** 18:00	0.20	0.79	0.74
P. abies	**7** 24:00	0.15	0.78	0.78
P. abies	**8** 6:00	0.20	0.81	0.71
P. abies	**9** 12:00	0.30	0.78	0.61
E. sylvaticum	**1** 12:00			
E. sylvaticum	**2** 18:00	0.25	0.80	0.67
E. sylvaticum	**3** 24:00	0.17	0.82	0.79
E. sylvaticum	**4** 6:00	0.22	0.81	0.75
E. sylvaticum	**5** 12:00	0.25	0.80	0.71
E. sylvaticum	**6** 18:00	0.23	0.82	0.82
E. sylvaticum	**7** 24:00	0.18	0.82	0.82
E. sylvaticum	**8** 6:00	0.19	0.81	0.81
E. sylvaticum	**9** 12:00	0.23	0.81	0.81
G. dryopteris	**1** 12:00	0.84	0.75	0.51
G. dryopteris	**2** 18:00	0.38	0.78	0.67
G. dryopteris	**3** 24:00	0.23	0.82	0.74
G. dryopteris	**4** 6:00	0.23	0.80	0.64
G. dryopteris	**5** 12:00	0.27	0.81	0.65
G. dryopteris	**6** 18:00	0.27	0.80	0.66
G. dryopteris	**7** 24:00	0.15	0.83	0.75
G. dryopteris	**8** 6:00	0.16	0.83	0.66
G. dryopteris	**9** 12:00	0.58	0.77	0.18
L. clavatum	**1** 12:00	0.53	0.79	0.38
L. clavatum	**2** 18:00	0.28	0.77	0.67
L. clavatum	**3** 24:00	0.20	0.77	0.73
L. clavatum	**4** 6:00	0.18	0.79	0.63
L. clavatum	**5** 12:00	0.28	0.78	0.58
L. clavatum	**6** 18:00	0.28	0.77	0.61
L. clavatum	**7** 24:00	0.24	0.78	0.72
L. clavatum	**8** 6:00	0.45	0.79	0.60
L. clavatum	**9** 12:00	0.28	0.77	0.54
C. majalis	**1** 12:00	0.69	0.76	0.56
C. majalis	**2** 18:00	0.69	0.76	0.50
C. majalis	**3** 24:00	0.61	0.80	0.80
C. majalis	**4** 6:00	0.25	0.79	0.73
C. majalis	**5** 12:00	0.34	0.79	0.71
C. majalis	**6** 18:00	0.40	0.78	0.78
C. majalis	**7** 24:00	0.30	0.80	0.80

C. majalis	**8** 6:00	0.21	0.82	0.82
C. majalis	**9** 12:00	0.35	0.80	0.80
A. monticola	**1** 12:00	0.38	0.80	0.37
A. monticola	**2** 18:00	0.67	0.76	0.31
A. monticola	**3** 24:00	0.17	0.82	0.76
A. monticola	**4** 6:00	0.17	0.83	0.69
A. monticola	**5** 12:00	0.13	0.83	0.72
A. monticola	**6** 18:00	0.13	0.83	0.69
A. monticola	**7** 24:00	0.12	0.83	0.77
A. monticola	**8** 6:00	0.11	0.83	0.71
A. monticola	**9** 12:00	0.33	0.81	0.58
M. bifolium	**1** 12:00	0.82	0.71	0.31
M. bifolium	**2** 18:00	0.35	0.72	0.64
M. bifolium	**3** 24:00	0.31	0.76	0.71
M. bifolium	**4** 6:00	0.37	0.75	0.59
M. bifolium	**5** 12:00	0.50	0.76	0.61
M. bifolium	**6** 18:00	0.50	0.76	0.55
M. bifolium	**7** 24:00	0.25	0.78	0.70
M. bifolium	**8** 6:00	0.30	0.78	0.61
M. bifolium	**9** 12:00	0.79	0.71	0.38
Sphagnum sp	**1** 12:00	0.72	0.72	0.61
Sphagnum sp	**2** 18:00	0.43	0.70	0.68
Sphagnum sp	**3** 24:00	0.31	0.73	0.81
Sphagnum sp	**4** 6:00	0.37	0.77	0.68
Sphagnum sp	**5** 12:00	0.61	0.75	0.52
Sphagnum sp	**6** 18:00	0.39	0.71	0.71
Sphagnum sp	**7** 24:00	0.32	0.77	0.77
Sphagnum sp	**8** 6:00	0.26	0.77	0.77
Sphagnum sp	**9** 12:00		0.76	0.76
P. juniperinum	**1** 12:00	0.61	0.80	0.53
P. juniperinum	**2** 18:00	0.34	0.81	0.68
P. juniperinum	**3** 24:00	0.25	0.81	0.74
P. juniperinum	**4** 6:00	0.26	0.82	0.70
P. juniperinum	**5** 12:00	0.36	0.80	0.30
P. juniperinum	**6** 18:00	0.36	0.78	0.65
P. juniperinum	**7** 24:00	0.24	0.81	0.74
P. juniperinum	**8** 6:00	0.33	0.80	0.70
P. juniperinum	**9** 12:00	0.55	0.77	0.43
B. pendula	**1** 12:00	0.47	0.81	0.66
B. pendula	**2** 18:00	0.29	0.81	0.61
B. pendula	**3** 24:00	0.22	0.85	0.83
B. pendula	**4** 6:00	0.27	0.81	0.77
B. pendula	**5** 12:00	0.29	0.84	0.76
B. pendula	**6** 18:00	0.30	0.85	0.85
B. pendula	**7** 24:00	0.17	0.84	0.84
B. pendula	**8** 6:00	0.29	0.85	0.85
B. pendula	**9** 12:00	0.16	0.83	0.83
S. aucuparia	**1** 12:00	0.76	0.73	0.54
S. aucuparia	**2** 18:00	0.28	0.76	0.65
S. aucuparia	**3** 24:00	0.19	0.79	0.71
S. aucuparia	**4** 6:00	0.18	0.78	0.62
S. aucuparia	**5** 12:00	0.21	0.81	0.67
S. aucuparia	**6** 18:00	0.21	0.80	0.57

S. aucuparia	**7** 24:00	0.14	0.81	0.73
S. aucuparia	**8** 6:00	0.13	0.82	0.69
S. aucuparia	**9** 12:00	0.72	0.76	0.45
V. myrtillus	**1** 12:00	0.69	0.78	0.42
V. myrtillus	**2** 18:00	0.34	0.78	0.64
V. myrtillus	**3** 24:00	0.26	0.80	0.72
V. myrtillus	**4** 6:00	0.25	0.78	0.68
V. myrtillus	**5** 12:00	0.29	0.72	0.42
V. myrtillus	**6** 18:00	0.29	0.78	0.65
V. myrtillus	**7** 24:00	0.29	0.78	0.71
V. myrtillus	**8** 6:00	0.40	0.80	0.60
V. myrtillus	**9** 12:00	0.59	0.75	0.34
V. vitis-idaea	**1** 12:00	0.66	0.82	0.56
V. vitis-idaea	**2** 18:00	0.62	0.82	0.75
V. vitis-idaea	**3** 24:00	0.57	0.83	0.81
V. vitis-idaea	**4** 6:00	0.58	0.82	0.76
V. vitis-idaea	**5** 12:00	0.47	0.83	0.73
V. vitis-idaea	**6** 18:00	0.53	0.83	0.83
V. vitis-idaea	**7** 24:00	0.43	0.83	0.83
V. vitis-idaea	**8** 6:00	0.41	0.83	0.83
V. vitis-idaea	**9** 12:00	0.41	0.83	0.83
R. tomentosum	**1** 12:00	0.37	0.80	0.43
R. tomentosum	**2** 18:00	0.53	0.77	0.33
R. tomentosum	**3** 24:00	0.25	0.79	0.70
R. tomentosum	**4** 6:00	0.26	0.80	0.66
R. tomentosum	**5** 12:00	0.30	0.79	0.72
R. tomentosum	**6** 18:00	0.30	0.80	0.71
R. tomentosum	**7** 24:00	0.24	0.78	0.76
R. tomentosum	**8** 6:00	0.27	0.79	0.75
R. tomentosum	**9** 12:00	0.25	0.78	0.68
P. aphthosa	**1** 12:00	0.66		
P. aphthosa	**2** 18:00	0.59		
P. aphthosa	**3** 24:00	0.67		
P. aphthosa	**4** 6:00	0.72		
P. aphthosa	**5** 12:00	0.69		
P. aphthosa	**6** 18:00	0.30	0.70	0.70
P. aphthosa	**7** 24:00	0.20	0.72	0.72
P. aphthosa	**8** 6:00	0.19	0.74	0.74
P. aphthosa	**9** 12:00	0.28	0.72	0.72
C. islandica	**1** 12:00	0.80	0.70	0.70
C. islandica	**2** 18:00	0.80		
C. islandica	**3** 24:00	0.58	0.57	0.58
C. islandica	**4** 6:00	0.50	0.68	0.74
C. islandica	**5** 12:00	0.82		
C. islandica	**6** 18:00	0.82		0.51
C. islandica	**7** 24:00	0.41	0.71	0.64
C. islandica	**8** 6:00	0.31	0.72	0.63
C. islandica	**9** 12:00	0.82		

Table S3: original data set (AZ/VAZ, Φ_{PSII} and FV/Fm) at each of the nine different measuring time points (0-48 h).

	CumPFD		Tmean	
Species	Highest R^2	Time Window	Highest R^2	Time Window
P. aphthosa	0.77	24	0.74	24
C. islandica	0.79	10	0.76	6
P. juniperinum	0.61	1	0.33	1
Sphagnum sp.	0.87	4	0.42	2
E. sylvaticum	0.77	2	0.55	1
G. dryopteris	0.92	4	0.25	19
L. clavatum	0.17	2	0.11	24
P. sylvestris	0.79	14	0.67	6
P. abies	0.54	12	0.49	7
A. monticola	0.88	10	0.65	4
C. majalis	0.82	16	0.62	8
M. bifolium	0.73	1	0.51	16
B. pendula	0.55	5	0.25	1
S. aucuparia	0.89	1	0.29	19
V. myrtillus	0.42	1	0.16	24
V. vitis-idaea	0.74	24	0.19	24
R. tomentosum	0.63	12	0.51	7

Table S4: Highest value of Pearson correlation coefficients (R^2) of the relationship between AZ/VAZ and cumulative photon dose (CumPFD) previously received by the leaf during a certain time interval (from 1 to 24 h, $CumPFD_1$ to $CumPFD_{24}$) (first column), or the mean temperature (Tmean) over the same time intervals (third column). Second and fourth column represent the time interval at which the strongest correlations were observed.
